# The Use of Artificial Intelligence to Detect Malignant Skin Lesions

**DOI:** 10.1016/j.mcpdig.2024.04.003

**Published:** 2024-04-05

**Authors:** Sofia Haddadin, Latha Ganti

**Affiliations:** a Trinity Preparatory School, Winter Park, FL; b University of Central Florida, Orlando, FL; c Brown University, Providence, RI; d Orlando College of Osteopathic Medicine, Winter Garden, FL

**Article Highlights**

• Artificial intelligence is a powerful tool that can be used to improve diagnostic capability in medicine.
• Using data-driven algorithms, tools are available on the market today to improve the diagnostic capability in evaluating skin lesions.
• There are several technologies that specifically focus on the detection of lesions suspicious for melanoma, one of the deadliest skin cancers.
• This article will review several of the abovementioned technologies currently available.

Artificial intelligence (AI) has emerged as a transformative tool in various medical fields. It is especially prevalent in dermatology, particularly in the early detection and diagnosis of skin cancer. This review will focus on AI tools to detect melanomas, the deadliest kind. The tools reviewed were identified from a Google search using the search term *technologies to detect melanomas* and from PubMed using the search term *artificial intelligence [title] AND melanoma [title]*. This review presents a slice of commercial tools available but is not a comprehensive review of all algorithms or teledermatology or AI methods that exist. As this is a rapidly evolving field, there may be even more information available by the time this work is published.

Skin cancer, one of the most common types of cancer globally, presents a significant public health challenge. In the United States, 1 in 5 will develop skin cancer by the age of 70 years, and more than 2 people die every hour from it.^1^, ^2^, ^3^, ^4^ Skin cancer can be divided into melanoma and nonmelanoma types; all types are associated with exposure to ultraviolet (UV) radiation from the sun.^5^^,^^6^ Basal cell and squamous cell carcinomas are the most common types of nonmelanoma lesions. While all 3 types originate in the epidermis, melanomas are the most invasive and, thus, considered the deadliest form of skin cancer.^7^ In the United States, almost 5.4 million people are diagnosed with skin cancer every year, with an early-detection survival rate of 97%.^1^ However, it drops to approximately 14% if detected in the late stages.^8^ Thus, early detection is crucial for successful treatment outcomes, yet it remains a complex task owing to the subtle and varied presentation of skin lesions. This is where artificial intelligence (AI) can be a valuable tool.

Artificial intelligence is the development of computer systems able to perform tasks that normally require human intelligence. These can include things such as speech recognition, translation between languages, and visual perception.^9^, ^10^, ^11^ AI can analyze large amounts of patient data, leading clinicians to make informed decisions about patient care quicker, thereby to improved patient outcomes. Artificial intelligence has been used in health care since the early 1970s when the field of AI was only 15 years old. Years later, in the early 2000s, AI was adapted to be used in the field of dermatology to assess the likelihood of a skin lesion being malignant.^12^^,^^13^ AI can use machine learning from dermoscopic images to accurately identify and classify skin lesions as benign or malignant, which in turn increases patient survival rates.^9^

## Review

Because of such a high rate of skin cancer diagnoses, AI tools have been developed by various companies to turn the tide and increase survival rates. Although there are many different tools that can be used, this review will focus on just 4 of the currently available pieces of technology derived from searching Google using the following string *technologies to detect melanomas* and from PubMed using *artificial intelligence [title] AND melanoma [title]*.

The first technology reviewed is Google DermAssist. This technology was developed using 16,114 deidentified clinical data and photographs from a 17-site teledermatology practice.^14^ Powered by deep learning–based skin evaluation software developed by Google Health, the application on the user's smart device asks them to upload 3 photos of the skin condition and answer questions that will be prompted by the program. This deep learning system was found to be noninferior to 6 dermatologists and superior to 6 primary care physicians and 6 nurse practitioners.^14^ A subsequent study demonstrated the technology to be significantly associated with higher agreement with reference diagnoses. The increase in diagnostic agreement for primary care physicians was 10%, from 48% to 58%; for nurse practitioners, the increase was 12% (46%-58%) (both *P*<.001).^15^ The study concluded that AI assistance was associated with improved diagnoses by both clinician types for 1 in every 8 to 10 cases and resulted in a decrease in biopsies and referrals.

The software uses AI to assist in diagnosing skin conditions and has been integrated into Google Lens, allowing users to search for skin conditions.^16^^,^^17^ Within a minute, the user receives results of possible matching skin conditions along with important information about each. Google DermAssist is not meant to replace a clinician diagnosis. However, it can help the user gain insight into their skin condition, which may lead them to obtain further information from their dermatologist.

DermAssist has been CE-marked as a class 1 medical device in the European Economic Area, indicating it meets high health and safety requirements.^16^ However, at the time of this writing, DermAssist was not available in the United States and the Food and Drug Administration has not evaluated this tool for safety or efficacy. Although it is intended for informational purposes and not for providing medical diagnoses, there is potential for misuse, especially as a consumer-facing tool.

The second technology is MelaFind, which has been around for more than a decade, approved in 2011.^18^ MelaFind is a multispectral instrument that uses automatic image analysis to classify lesions by morphologic disorganization and recommends which lesions should be biopsied.^19^ The device was developed to evaluate suspicious skin lesions and determine whether pigmented skin lesions are melanoma or benign. The 2011 multicenter prospective trial consisted of 1383 patients with a median age of 47 years, who had 1831 pigmented lesions.^19^ A sensitivity of 98% and specificity of 10% were reported, with a corresponding positive predictive value of 11.7% and negative predictive value of 98.1%.

MelaFind is not able to detect all possible melanomas, with its range being cancers from 0.2 to 2.2 cm. The device is noninvasive and consists of a handheld imager, a monitor for displaying results, a computer connected to the imager, and a removable media for storing MelaFind results and images. Once the lesion is scanned, the photograph is compared with a database of 10,000 skin biopsies. The output is then either a “yes” or “no” answer (for whether to do a biopsy) based on the degree of morphologic disorganization of the lesion.^20^

MelaFind has excellent sensitivity, ranging from 94% to 98%, but poor specificity, ranging from 8% to 44%.^18^, ^19^, ^20^, ^21^ An online survey of dermatologists who were presented 130 clinical images with and without the aid of MelaFind found sensitivity to increase from 69.5% to 79% (1-sided *P*<.00001).^18^ They also noted that MelaFind performed better than a dermatologist alone. In a study of 111 patients with 360 atypical pigmented skin lesions examined with MelaFind, among all lesions biopsied (n=113), the sensitivity and specificity of MelaFind was 100%.^20^

The third technology is Nevisense.^22^^,^^23^ The diagnostic support tool uses impedance spectrospcopy to measure skin cell size, shape, and position beneath the skin’s surface, checking for abnormalities. Nevisense is indicated to be used on skin lesions between 2 and 20 mm in diameter. The tool is a small handheld device connected to a visual monitor that displays results.

A multicenter, prospective, blinded study was conducted at 5 American and 17 European investigational sites to assess the effectiveness and safety of Nevisense in the distinction of benign lesions from melanoma. Over 1900 lesions were evaluated, including 265 melanomas. Nevisense was found to have a 96.6% sensitivity,^24^ suggesting the technology is an accurate and safe device to support clinical decision making. Nevisense technology has also reported positive results in the detection of eczema versus dry skin.^25^

The fourth technology is the Veriskin Truscore product.^26^ This noninvasive handheld device allows the physician to see cutaneous hemodynamic results in real-time, reducing the testing-to-diagnosis time frame. The tool was developed to be used as a decision-support tool for physicians. It is lightweight and compact, weighs 130 g, and is made of high-impact polymers for high durability. The device was additionally featured by the National Institute of Health, Novo Engineering, the National Cancer Institute, TEDx SanDiego, and others. The product is not currently for sale at the time of writing this article.

## Discussion

Up to 20% of primary care visits are for skin complaints.^27^, ^28^, ^29^, ^30^ Melanoma will claim approximately 8000 lives in 2024,^31^ almost all due to late-stage diagnosis. Melanoma in situ with a Breslow thickness of 1 mm or thinner has a 5-year survival rate of 94%—contrast that to stage IV melanoma, which is 15% at 5 years,^32^^,^^33^ with most patients dying within 6 to 10 months.

The integration of AI in dermatologic practice can serve as a decision-support tool, augmenting the diagnostic accuracy of clinicians. AI algorithms, particularly convolutional neural networks, have been at the forefront of this revolution. These algorithms analyze dermatoscopic images, effectively learning from each case, thereby improving their diagnostic performance over time. Studies have shown that in some instances, AI-driven diagnostic systems can match or even surpass the diagnostic accuracy of human experts.

Artificial intelligence and machine learning in detecting skin cancers come with a plethora of benefits. Artificial intelligence has progressed to the point where it can successfully detect skin cancer earlier than traditional methods of identification.^34^ With machine learning, more images are uploaded to various databases; the technology will be able to accurately identify even more possibly cancerous lesions. Studies show that they can perform as well as or even better than dermatologists and assist in diagnosing skin cancers.^35^^,^^36^ It may even improve skin examinations by cataloging patient lesions and detecting and tracking lesions that concern care providers. Patients are able to inspect and send images of their skin condition(s) to their dermatologist from the comfort of their home by, for example, attaching a lens to their smartphone and uploading images through a secure portal. Artificial intelligence also allows for teledermatology, which greatly expands patient access. Instead of waiting weeks or possibly months to get the condition inspected, the patient gains peace of mind earlier. However, there is still an AI error, so a second check by an additional external source, preferably a human, is needed until all issues are resolved and the technology has been around for a long time. Currently, AI can only supplement the decision made by the dermatologist and provide a need to prompt further examination of the skin lesion in question.

However, there are still downsides to the technology that must be overcome to fully reap the benefits of it. With AI using image-based machine learning, altered or blurry photographs may impact and prohibit an accurate diagnosis. The misdiagnosis may prohibit or delay the patient from getting treatment for a possibly malignant skin lesion, leading to further issues. A solution to this would be to have the AI prompt the user to retake the photograph if it is not able to give an accurate diagnosis. Another barrier the AI must overcome is the lack of patient trust in the technology, including concerns about the regulations and laws pertaining to the safe use of AI in health care. Moreover, 63% of patients surveyed reported being unwilling to trust AI in health care generally.^37^ The lack of trust is a barrier that will be overcome with time. The longer AI is present in the health care system, and people see how it is being impacted by the technology, the lower the lack of trust will be. In addition, dermatologists are concerned with AI disrupting the clinician-patient relationship. If patients are self-diagnosing through AI tools at home, they are not going into the dermatologist’s office, causing a strain in the relationship. Another factor that leads to this is the fact that if AI were solely diagnosing patients, clinicians would not be aware of how this diagnosis was made because the process is not observable. This led to significant criticism of the technology for diagnostic purposes and the risk of legal challenges if the diagnosis is solely based on the AI conclusion.

Despite this, there has been a big push for transparency and data handling when it comes to AI in the health care setting, specifically in diagnostics. Another weakness in AI that must be overcome is having a trained algorithm system that identifies skin conditions on all skin colors. Most current AI algorithms are trained solely on Caucasian or Asian patients. If we expand the scanning field to other skin colors in the form of early screenings, then the algorithm will be trained on a broader range of images from different ethnicities. This will lead to increased health equity and diversity when it comes to AI in detecting skin cancers.

## Conclusion

The use of AI technology in detecting skin cancers is vital in evolving the health care field, specifically dermatology. Early-detection survival rates are drastically higher than late detection survival rates. Machine learning, a subset of AI, can be used to train the algorithms to be able to identify a variety of malignant skin lesions as more images are uploaded to databases. Many tools and algorithms have been developed to detect skin cancer. Each tool showcases the power of digital health and a glimpse into the near future of health care.

## Potential Competing Interests

The authors report no competing interests.
